# MRI of Uveal Melanoma

**DOI:** 10.3390/cancers11030377

**Published:** 2019-03-17

**Authors:** Teresa A. Ferreira, Lorna Grech Fonk, Myriam G. Jaarsma-Coes, Guido G. R. van Haren, Marina Marinkovic, Jan-Willem M. Beenakker

**Affiliations:** 1 Department of Radiology, Leiden University Medical Centre, Albinusdreef 2, 2333 ZA Leiden, The Netherlands; L.grech_fonk@lumc.nl (L.G.F.); M.G.Jaarsma@lumc.nl (M.G.J.-C.); G.R.van_Haren@lumc.nl (G.G.R.v.H.); J.W.M.Beenakker@lumc.nl (J.-W.M.B.); 2 Department of Ophthalmology, Leiden University Medical Centre, Albinusdreef 2, 2333 ZA Leiden, The Netherlands; M.Marinkovic@lumc.nl

**Keywords:** uveal melanoma, MRI, diffusion-weighted imaging, perfusion-weighted imaging

## Abstract

Uveal Melanoma (UM) is the most common primary malignant ocular tumor. The high soft tissue contrast and spatial resolution, and the possibility of generating 3D volumetric and functional images, make Magnetic Resonance Imaging (MRI) a valuable diagnostic imaging technique in UM. Current clinical MRI protocols, however, are not optimized for UM and therefore lack the quality for accurate assessments. We therefore developed a dedicated protocol at a 3 Tesla MRI, using an eye coil, consisting of multi-slice 2D sequences, different isotropic sequences and diffusion and perfusion-weighted images. This protocol was prospectively evaluated in 9 uveal melanoma patients. The multi-slice 2D sequences had the highest in-plane resolution, being the most suited for lesion characterization and local extension evaluation. The isotropic 3D Turbo-Spin Echo (TSE) sequences were the most suitable for accurate geometric measurements of the tumor and are therefore important for therapy planning. Diffusion and perfusion-weighted images aid in differentiating benign from malignant lesions and provide quantitative measures on tumor hemodynamics and cellularity, which have been reported to be effective in predicting and assessing treatment outcome. Overall, this dedicated MRI protocol provides high-quality imaging of UM, which can be used to improve its diagnosis, treatment planning, and follow-up.

## 1. Introduction

Uveal melanoma (UM) is the most common primary intraocular malignancy in adults, although with an incidence of about 6 cases per million per year [1,2]. UM arises in 85% of cases from the choroid, the remainder originating from the ciliary body or iris.

In the past, enucleation was the main treatment, but in the last decade(s) various eye- and vision-saving treatments have become available, with the purpose of achieving local tumor control, conserving the eye and useful vision. This is of significant benefit to the quality of life for many patients. These eye-preserving therapies include various forms of radiotherapy, such as episcleral brachytherapy, proton-beam radiotherapy and stereotactic radiotherapy [3,4].

The ideal imaging technique for the evaluation of UM needs to be capable of accurately delineating the limits of the tumor, of doing accurate measurements and of evaluating the presence of extrascleral extension, since these are the main determinants for the choice of the type of treatment, and in the case of radiotherapy for the radiotherapy planning. Additionally, noninvasive markers that can predict treatment response and prognosis are needed [5,6], in order to adjust the frequency and type of screening according to whether the patient is at high or low risk of developing disseminated disease, and because even if a biopsy is performed, it can be nonrepresentative, due to UM being heterogeneous in terms of chromosomal aberrations [7,8]. Finally, it should be able to assess tumor response to radiotherapy both in terms of size and internal structure at an early stage.

UM has traditionally been evaluated with ultrasound (US), fundoscopy, and fluorescein angiogram (FA). Imaging the eye with Magnetic Resonance Imaging (MRI) is a challenge due to eye motion and to the magnetic susceptibility effects at the air–bone interface. However, recent developments on MRI make it a promising diagnostic imaging modality in ophthalmology, due to its excellent soft tissue contrast and spatial resolution, as well as its possibility for generating functional images such as diffusion-weighted imaging (DWI) and perfusion-weighted imaging (PWI) [5,6,9]. UM is therefore more and more being evaluated with MRI [5,6,9,10,11]. The purpose of our study was to optimize the MRI technique of the globe and in particular of uveal melanomas.

## 2. Material and Methods

This single-center prospective study was carried out according to the Code of Ethics of the World Medical Association (Declaration of Helsinki) for experiments involving humans and in accordance with recommendations of the local Ethic Committee (CME LUMC, Leiden University Medical Center, Project number P16.186). Informed Consent was obtained from all individual participants. A multiparametric ocular MRI protocol—Study Protocol—was prospectively evaluated at a 3T MRI (wide bore Ingenia 3T, Philips Healthcare, Best, The Netherlands) on nine consecutive patients with the diagnosis of UM. All patients were examined by an ocular oncologist, and the final diagnosis was made based on fundoscopic, ultrasonographic and fluorescein angiographic imaging. Seven of the subjects were male. The median age of all subjects was 62 years (range 31–81). In 89% of the cases the lesion was in the right eye. Table 1 shows the data regarding patients’ sex, eye involved, American Joint Committee on Cancer (AJCC) tumor class [12], treatment and histology results in case of enucleation.

### 2.1. General MRI Setup

**Figure 1:**
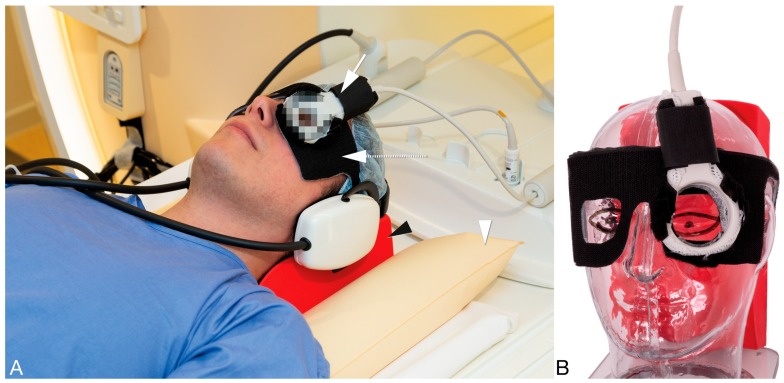
(**A**,**B**) Clinical setup for ocular MRI. (**A**) The patients’ head is supported by a radiotherapy head support (black arrowhead) which is stabilized by two sandbags (arrowhead). To aid an easy mounting of the eye coil (arrow) a pair of goggles (dashed arrow) is used on which the coil can be attached with Velcro. (**B**) Positioning of the eye coil and of the pair of goggles exemplified on a phantom to better show their relationship with respect to the eye.

A 4.7 cm surface receive coil (Philips Healthcare, Best, The Netherlands), in combination with the body-coil for transmit, was used to image the eye, in order to maximize the signal-to-noise (SNR) of the MRI images [13]. A pair of goggles, made from a thermoplastic material (Orfit industries, Wijnegem, Belgium), was constructed and covered with Velcro^®^ (Alfatex N.V., Deinze, Belgium), the latter permitting the attachment of the local receive coil to the goggles (Figure 1). Since the sensitivity of the coil decays as a function of the distance to the coil, image quality will depend strongly on the positioning of the coil and this system allows a good, easy and reproducible positioning of the eye coil [14]. Although a surface coil results in a higher SNR compared to the head coil, the absence of tight support of the head makes the scans much more vulnerable to motion. After preliminary experiments using different fixation methods, including cushions, sandbags and strapping the subject to the MRI table, a radiotherapy head support (MaxSupport^TM^ wide shaped, red variant, 117000 HSSETW, Medeo, Schöftland, Switzerland), supported by sandbags proved to be the optimal balance between stability, patient comfort and ease of use. To mitigate eye motion-related artefacts in ocular MRI different methods have been proposed, such as cued blinking approaches [15,16]. These methods, however, rely on modifications to the MRI scanner and/or MR sequences, which are difficult to use in clinical practice. We therefore decided to make no modifications to the conventional setup and only ask patients to close their eyes and to try to minimize their eye movement.

### 2.2. MRI Sequences

The purpose of our study was to design a MRI protocol optimized for the evaluation of uveal melanomas. The requirements for imaging in UM include: (1) origin of the lesion. (2) dome, mushroom or lentiform shape. (3) local extension, in particular sclera, extrascleral, or ciliary body invasion. (4) solid or necrotic structure. (5) signal intensity on T1 and T2 weighted imaging sequences (WI). (6) contrast enhancement. (7) DWI characteristics. (8) PWI with evaluation of the Time Intensity Curve (TIC). (9) dimensions–tumor prominence and largest basal tumor diameter (LBD). The tumor prominence is the tumor thickness and the sclera thickness was included in our series since in brachytherapy a radioactive source is sutured to the episcleral surface overlying the tumor. (10) presence of retinal detachment.

**Figure 2:**
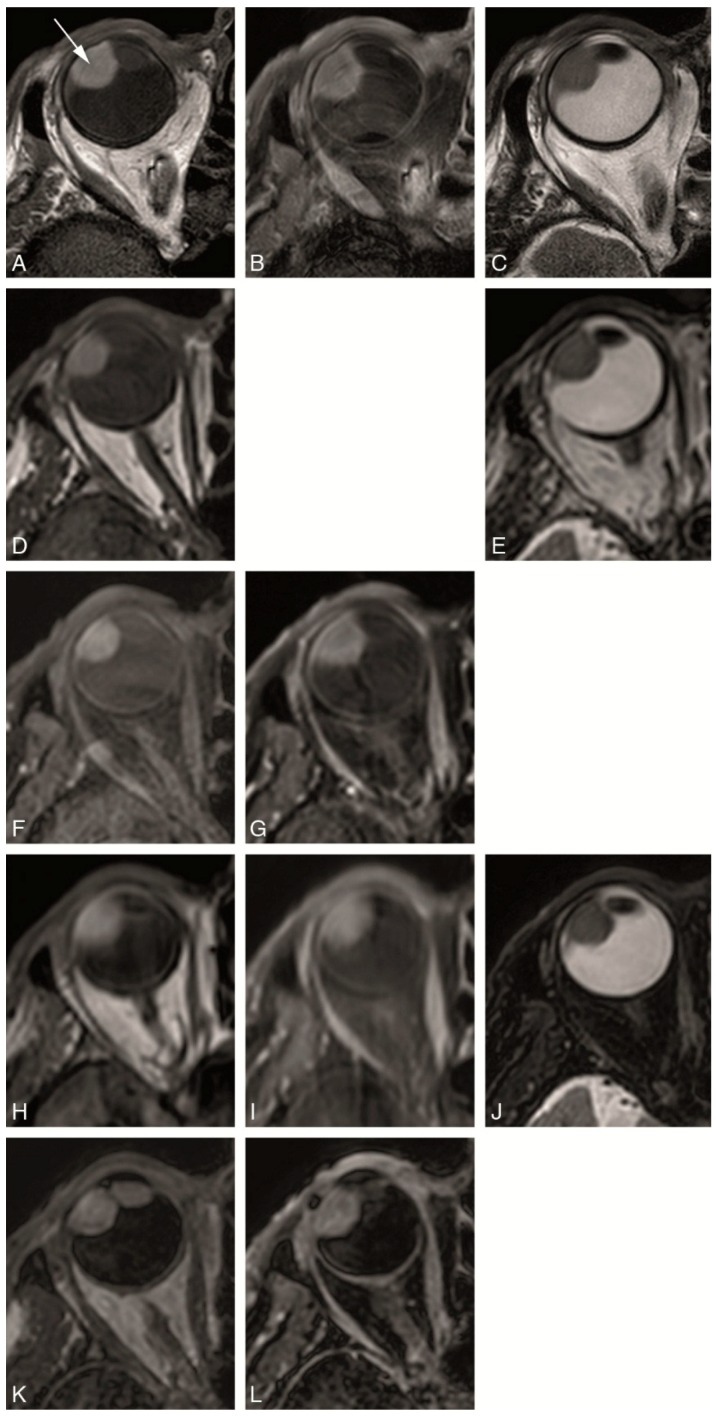
(**A**–**L**) Study protocol: Multiple anatomical axial MRI sequences in a patient with a uveal melanoma of the right eye (arrow). (**A**) MS 2 mm T1. (**B**) MS 2 mm enhanced T1 with fat signal suppression. (**C**) MS 2 mm T2. (**D**) MS 1 mm T1. (**E**) MS 1 mm T2. (**F**) MS 1 mm T1 with fat signal suppression. (**G**) MS 1 mm enhanced T1 with fat signal suppression. (**H**) 3D TSE 1 mm T1. (**I**) 3D TSE 1 mm enhanced T1 with fat signal suppression. (**J**) 3D TSE 0.8 mm T2 with fat signal suppression. (**K**) 3D TFE 0.8 mm T1. (**L**) 3D TFE PROSET 0.8 mm enhanced T1 with fat signal suppression.

**Figure 3:**
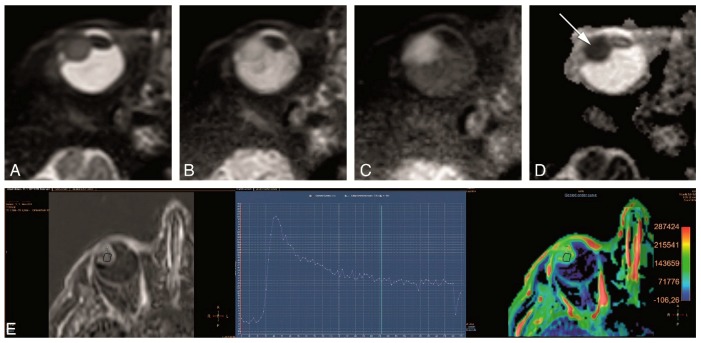
(**A**–**E**). Study protocol: Functional axial MRI sequences in the same patient as in Figure 2. (**A**–**D**) TSE DWI, with b values of 0 s/mm^2^ (**A**), 400 s/mm^2^ (**B**), 800 s/mm^2^ (**C**) and Apparent Diffusion Coefficient (ADC) (**D**). Restricted diffusion in the uveal melanoma (arrow). (**E**) Dynamic Contrast-Enhanced (DCE) with a good quality, no movement artefacts, showing a wash-out TIC pattern.

Multiple MRI sequences for imaging the eye had been developed previously by imaging eyes of healthy volunteers to find the optimal balance between SNR, field-of-view (FOV), minimal artefacts and scan time. These sequences had subsequently been evaluated on four UM patients to optimize the scans in terms of tumor contrast. A multiparametric ocular MRI protocol—Study Protocol, consisting both of anatomical and functional sequences, was then developed and subsequent prospectively evaluated in nine consecutive patients with the diagnosis of UM. This Study Protocol is described below and summarized in Table 2 and Figure 2 and Figure 3.

### 2.3. Anatomical MRI Sequences

The anatomical sequences of our Study Protocol are shown in Figure 2 and described in Table 2. We evaluated two different types of anatomical sequences. On one hand, multi-slice (MS) sequences with a slice thickness of 2 mm and a high in-plane resolution of approximately 0.5 mm were performed, which are mainly important for the evaluation of tumor origin and extension. These MS 2 mm sequences, due to their relatively thick slices, prevent multiplanar reconstructions being considered 2D sequences. On the other hand, isotropic sequences were performed, with an isotropic resolution of approximately 1 mm and therefore allowing multiplanar reconstructions, needed for accurate measurements. To assess the optimal sequence to acquire a 3D MRI of the eye three different isotropic sequences were tested, namely MS sequences with 1 mm slices, 3D turbo spin echo (TSE) sequences and 3D turbo field echo (TFE) sequences. While on a 3D-sequence the complete volume is acquired simultaneously, on a MS sequence multiple adjacent thin 2D slices are acquired. Furthermore, for the 3D sequences both gradient echo and spin echo techniques can be used. The gradient echo sequences allow for a slightly higher resolution per imaging time than spin echo sequences, but spin echo sequences are less affected by the magnetic field inhomogeneities caused by the tissue-air interfaces around the globe. Both the MS 2 mm 2D and the isotropic sequences included T1-WI and T2-WI sequences, with or without fat suppression, and T1-WI sequences after contrast medium administration with fat suppression. Although ideally all different combinations would have been evaluated, only a representative subset, listed in Table 2 and Figure 2, was acquired, to limit the total scan time to less than 45 min.

The anatomical sequences of our Study Protocol are shown in Figure 2 and described in Table 2. We evaluated two different types of anatomical sequences. On one hand, multi-slice (MS) sequences with a slice thickness of 2 mm and a high in-plane resolution of approximately 0.5 mm were performed, which are mainly important for the evaluation of tumor origin and extension. These MS 2 mm sequences, due to their relatively thick slices, prevent multiplanar reconstructions being considered 2D sequences. On the other hand, isotropic sequences were performed, with an isotropic resolution of approximately 1 mm and therefore allowing multiplanar reconstructions, needed for accurate measurements. To assess the optimal sequence to acquire a 3D MRI of the eye three different isotropic sequences were tested, namely MS sequences with 1 mm slices, 3D turbo spin echo (TSE) sequences and 3D turbo field echo (TFE) sequences. While on a 3D-sequence the complete volume is acquired simultaneously, on a MS sequence multiple adjacent thin 2D slices are acquired. Furthermore, for the 3D sequences both gradient echo and spin echo techniques can be used. The gradient echo sequences allow for a slightly higher resolution per imaging time than spin echo sequences, but spin echo sequences are less affected by the magnetic field inhomogeneities caused by the tissue-air interfaces around the globe. Both the MS 2 mm 2D and the isotropic sequences included T1-WI and T2-WI sequences, with or without fat suppression, and T1-WI sequences after contrasting medium administration with fat suppression. Although ideally all different combinations would have been evaluated, only a representative subset, listed in Table 2 and Figure 2, was acquired, to limit the total scan time to less than 45 min.

Preliminary evaluations in healthy subjects showed a different susceptibility to fold-over artefacts for the different scans, resulting on different FOV for the different scans. To ease the planning, however, a single FOV, 80 × 80 × 40 mm^3^, was set for most of the 3D scans and additional oversampling was added to prevent fold-over.

As the isotropic sequences allow for retrospective reformatting in all directions they were acquired on the axial plane non-angulated. In contrast, the MS 2 mm 2D sequences were acquired perpendicular to the main axis of the tumor, to allow for an optimal discrimination between the tumor, retina-choroid and sclera.

Given that the posterior aspect of the globe is surrounded by orbital fat, attention had to be given to the water-fat shift (WFS), since, depending on the direction of the WFS, the displaced fat might overlay the sclera/tumor or might lead to overestimating sclera thickness. Especially for the MS 2 mm 2D sequences, special care had to be given to the WFS during planning, since this scan has a relatively high WFS of 2.3 pixels whose direction depends on the, patient specific, angulation of the slice.

### 2.4. Functional MRI Sequences

The functional sequences of our Study Protocol, shown in Figure 3 and described in Table 2, included DWI and PWI.

The tumor microstructure was assessed through DWI. Our preliminary evaluation on the first group of four UM patients showed that a diffusion weighting of b = 1000 s/mm^2^, commonly used for neuro-imaging, resulted in a too strong attenuation of the signal from the tumor. We therefore evaluated two different levels of diffusion weighting, 400 s/mm^2^ and 800 s/mm^2^. The DWI acquisition was acquired in the same orientation as the MS 2 mm 2D sequences, although at a lower resolution, using a non-Echo Planar Imaging (EPI) TSE readout, since the EPI readout is too sensitive to B0-inhomogeneities, prevalent in the orbit.

To assess the tumor perfusion, a Dynamic Contrast-Enhanced (DCE) scan was included in the protocol, in which multiple images are acquired sequentially before, during and after intravenous administration of 0.1 mmol/kg gadoterate meglumine (gd-DOTA, DOTAREM, Guerbet, Roissy CdG Cedex, France). To achieve an increased temporal resolution key-hole imaging is often implemented [17], in which only the central part of k-space, which encodes for most of the contrast of the image, is acquired for all dynamics, and the peripheral k-space is only acquired in the first or last dynamic and subsequently used as a reference for all dynamics. Although the resulting increase in temporal resolution is certainly beneficial to assess inflow of the contrast agent, the sharing of k-space data makes the images very susceptible to eye motion, since a drift in gaze direction between the reference acquisition and the other acquisitions, will result in significant motion artefacts in all images. We therefore employed the TWIST (Time-resolved angiography With Stochastic Trajectories) methodology [18], in which the acquisition of the peripheral k-space is split between adjacent dynamics. As a result, the time difference between the central and peripheral k-space data is substantially reduced, resulting in a significant reduction of eye motion-related artefacts.

### 2.5. Evaluation

Images were evaluated by a Neuro and Head and Neck Radiologist with more than 20 years of experience and by an Ophthalmic MRI Specialist with 7 years of experience. Image quality, contrast and 3D geometrical accuracy were evaluated. The final decision was achieved by consensus.

## 3. Results

Despite the challenges of susceptibility artefacts and eye motion when imaging the eye with MRI, multiparametric MRI of the eye is feasible, good quality images can be obtained and MRI can be used for the evaluation of UM. The clinical diagnosis of UM was confirmed by histology on all the four enucleated cases. Table 3 shows the results of the evaluation of the several anatomical sequences of the Study Protocol. The in-plane image quality was evaluated for general image quality, contrast, identification of the sclera and of the tumor limits and differentiating the tumor from retinal detachment. Furthermore, the 3D sequences were evaluated regarding geometrical accuracy and identification of the sclera.

**Figure 4:**
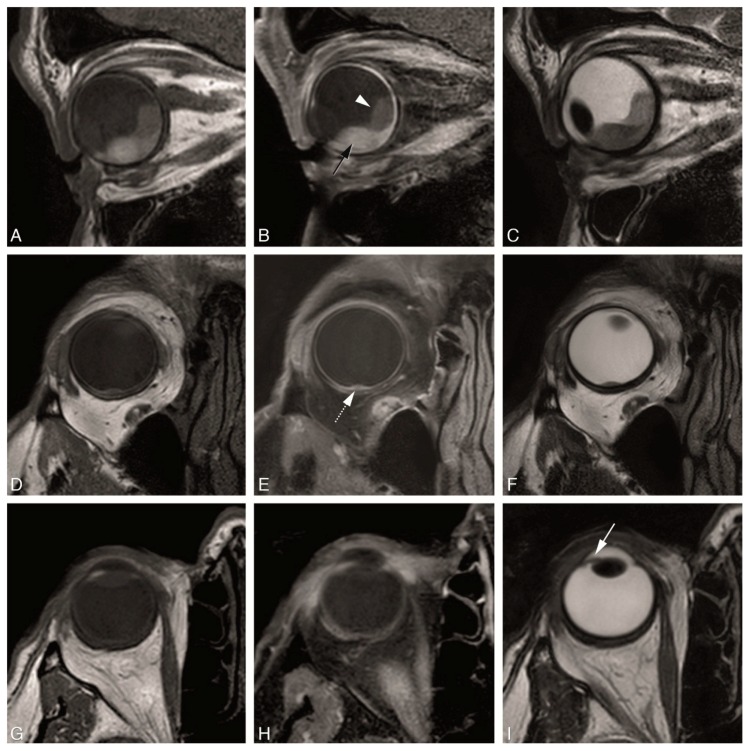
(**A**–**I**) MS 2 mm sequences in three different patients. (**A**–**C**)—MS 2 mm sagittal T1 (**A**), enhanced sagittal T1 with fat signal suppression (**B**) and sagittal T2 (**C**), in a patient with a large choroidal melanoma of the right eye (black arrow), with retinal detachment (arrowhead) associated. (**D**–**F**) MS 2 mm axial oblique T1 (**D**), enhanced axial oblique T1 with fat signal suppression (**E**) and axial oblique T2 (**F**), in a patient with a small choroidal melanoma of the right eye (dashed arrow). (**G**–**I**) MS 2 mm axial T1 (**G**), enhanced axial T1 with fat signal suppression (**H**) and axial T2 (**I**), in a patient with a small iris melanoma of the right eye (arrow). Notice that the MS 2 mm sequences have a high spatial resolution and therefore good for the evaluation of very small melanomas (**D**–**F**,**G**–**I**), even located at the iris.

The MS 2 mm 2D sequences were the sequences with the highest in-plane resolution in our series (Figure 4). The in-plane image quality of the MS 2 mm 2D sequences was always good or very good, while the in-plane image quality from the isotropic sequences was variable ranging from insufficient to very good (Table 3). They are therefore most suited to evaluate details such as normal eye anatomy, differentiating the layers of the globe wall and for a better delineation of the tumor boundaries. Regarding UM, this is needed for the evaluation of the layer of origin of the tumor, important for the differential diagnosis, and for the evaluation of UM extension, in particular scleral invasion, extrascleral extension or invasion of the ciliary body. Moreover, the MS 2 mm 2D sequences were the most suitable for evaluating the borders and shape of the tumor. Even quite flat UM with a prominence of 1–2 mm could be characterized (Figure 4D–F). UM arising in small structures such as ciliary body and iris could be visualized well, as long as the sequences were planned perpendicular to the main axis of the tumor (Figure 4G–I).

**Figure 5:**
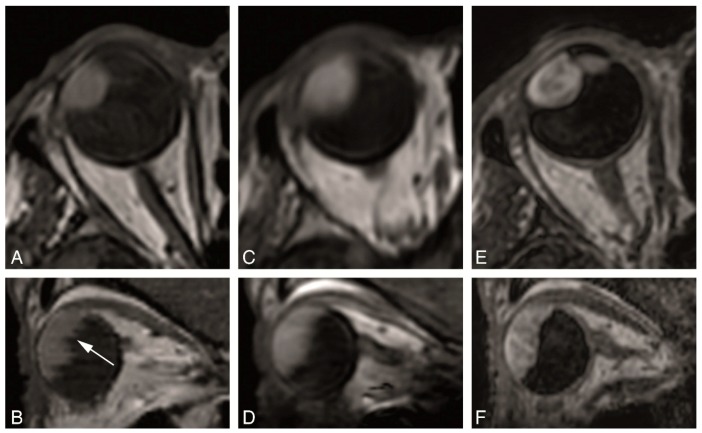
(**A**–**F**) MS 1 mm versus 3D sequences in a patient with a uveal melanoma of the right eye. (**A**,**B**) MS 1 mm axial T1 (**A**) and sagittal reconstruction (**B**). (**C**,**D**) 3D TSE 1 mm axial T1 (**C**) and sagittal reconstruction (**D**). (**E**,**F**) 3D TFE 0.8 mm axial T1 (**E**) and sagittal reconstruction (**F**). Notice the stripes and wall deformation on the 3D reconstructions from the MS 1 mm sequence (**B**) (arrow), a frequent problem encountered when there is eye motion. This effect is not present in the 3D reconstructions from the 3D TSE or 3D TFE sequences, although some mild blurring is visible in the 3D TSE reconstruction.

Multiplanar reconstructions are needed for evaluating the tumor in different planes, to assess tumor geometry, for accurate measurements [9] and also play a role in the assessment of sclera/extrascleral extension. Multiplanar reconstructions are not feasible with the MS 2 mm 2D sequences as the relatively thick slices prevent perpendicular reconstructions. Multiplanar reconstructions were possible with all isotropic sequences, but in general we noticed a better quality of the multiplanar reconstructions from the 3D sequences when compared to the MS 1 mm sequences (Figure 5). The geometrical accuracy from the 3D sequences was very good, while the geometrical accuracy from the MS 1 mm sequences ranged from sufficient to good (Table 3). Due to the relatively large acquisition time differences between subsequent slices in MS sequences, eye motion results in significant deformations, preventing accurate measurements (Figure 5B). 3D sequences are less sensitive to eye movement and therefore the reconstructions were of good quality, although small ghosting artefacts could be seen due to eye motion (Figure 5D,F).

**Figure 6:**
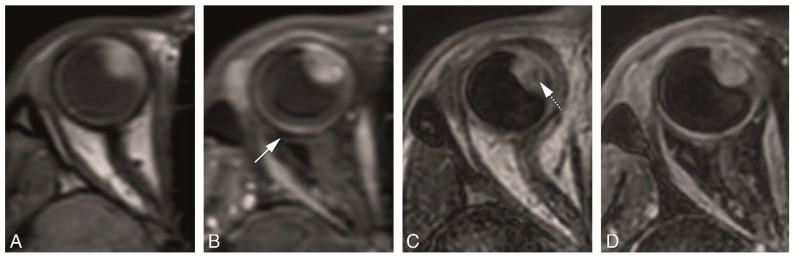
(**A**–**D**) 3D SE versus 3D GE sequences in two different patients. (**A**–**D**) 3D TSE 1 mm axial T1 (**A**), 3D TSE 1 mm enhanced axial T1 with fat signal suppression (**B**), 3D TFE 0.8 mm axial T1 (**C**) and 3D TFE 0.8 mm enhanced axial T1 with fat signal suppression (**D**), in a patient with a large uveal melanoma of the right eye. On one hand, on the SE sequences the outer limit of the sclera is better visualized (arrow), while on the GE sequences the outer limit of the sclera is more difficult to identify. On the other hand, the contrast resolution is better on the GE sequences, where a small area of necrosis is seen within the tumor (dashed arrow).

Comparison between 3D spin echo (SE) and 3D gradient echo (GE) sequences showed that the wall of the eye, in particular the outer limit of the sclera, is best identified on the SE sequences, while on the GE sequences, the outer limit of the sclera cannot be identified clearly. Contrast resolution on the other hand, was higher on the GE sequences, allowing better characterization of the tumor structure, in particular of tumor heterogeneity due to necrosis (Figure 6).

In our protocol the post-contrast T1-weighted scans were acquired with fat suppression, allowing for a better visualization of potential extra-scleral extension of the tumor. For pre-contrast T1 and T2 scans both fat-suppressed and unsuppressed scans were performed and have advantages. On one hand, in general pre-contrast T1 without fat suppression and T2 with fat suppression make infiltration of fat more conspicuous, but in the case of UM that will also depend on its melanotic content and therefore on its signal intensity on T1 and T2. On the other hand, a pre-contrast T1 with fat suppression allows for a better comparison with the post-contrast T1, also with fat suppression.

**Figure 7:**
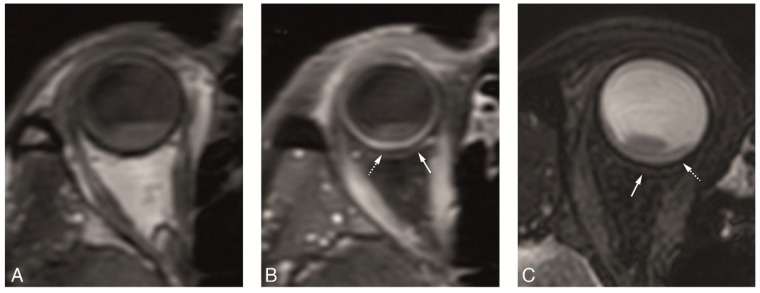

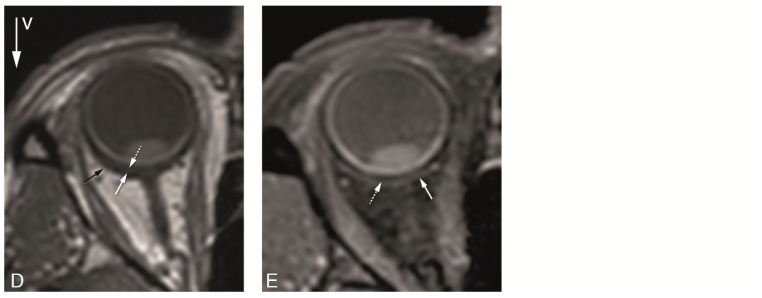
(**A**–**E**) Water-fat shift artefact and “extra layer” outside sclera. 3D TSE 1 mm T1 (**A**), 3D TSE 1mm enhanced T1 with fat signal suppression (**B**), 3D TSE 0.8 mm T2 with fat signal suppression (**C**), MS 1 mm T1 (**D**) and MS 1 mm enhanced T1 with fat signal suppression (**E**) in the same patient. The water-fat shift artefact is prominent on the MS sequence (**D**) (black arrow), seen as a signal void crescent area at the sclera-orbital fat interface, occurring in the frequency-encoding direction and therefore behind the sclera. It is important to be acquainted with this artefact not to misinterpret it as sclera which would overestimate the sclera thickness. The water-fat shift artefact is negligible on the 3D TSE sequence (**A**) and it is not present on the sequences with fat suppression (**B**,**C**,**E**). Notice, especially on the sequences with fat signal suppression (**B**,**C**,**E**), but also visible on the MS 1 mm T1 (**D**), the presence of a thin layer slight hyperintense on T1 and T2 (arrows), perhaps corresponding to the Tenon’s space or to a slight layer of fluid outside the globe, allowing a good identification of the outer limit of the sclera (dashed arrows).

The chemical shift artefact at the sclera-fat interface, originating from the wrong spatial encoding of fat protons in the frequency-encoding direction, is seen on the sequences without fat suppression, being removed with fat suppression (Figure 7). In our protocol, the water-fat shift artefact was more prominent on the MS sequences, compared to the 3D TSE sequences.

We noticed, especially on the sequences with fat signal suppression, but also on the MS 1 and 2 mm T1 and T2 sequences, the presence of a thin layer, located just adjacent to the globe, slight hyperintense on T1 and T2, allowing a good identification of the outer limit of the sclera (Figure 7), which was very helpful in measurements of tumor dimensions. This layer likely corresponds to the Tenon’s space or to a slight layer of fluid outside the globe, and can also be seen on ultrasound A-scan as a small dip in reflectivity between the tumor and the extraocular structures.

Identifying enhancement of the lesion is crucial to differentiate UM from hematoma for instance. Because of its melanin content, UM are frequently spontaneously hyperintense on T1, making it difficult to be sure it is enhancing just from the visual evaluation of the post-contrast series. For that the perfusion sequence is diagnostic and we did pre-contrast sequences also with fat suppression for an easier comparison.

**Figure 8:**
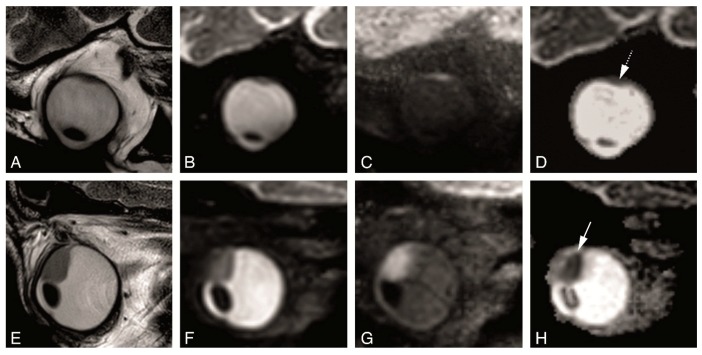
(**A**–**H**) TSE DWI in two different patients. (**A**–**D**) MS 2 mm sagittal oblique T2 (**A**), TSE DWI sagittal obliques with b values of 0 s/mm^2^ (**B**), 800 s/mm^2^ (**C**) and ADC (**D**). Restricted diffusion easy to appreciate in a small uveal melanoma (dashed arrow). (**E**-**H**) MS 2 mm sagittal oblique T2 (**E**), TSE DWI sagittal obliques with b values of 0 s/mm^2^ (**F**), 800 s/mm^2^ (**G**) and ADC (**H**). Clear restricted diffusion in the tumor (arrow).

Although it has been reported that the non-EPI TSE DWI is limited by poor SNR relative to the acquisition time [6], we found mostly a good quality, with restriction diffusion easy to appreciate even in small uveal melanomas (Figure 8). We noticed moreover that the DWI sequence with a b value of 400 s/mm^2^ was not as good as the one with a b value of 800 s/mm^2^ and of no additional value.

**Figure 9:**
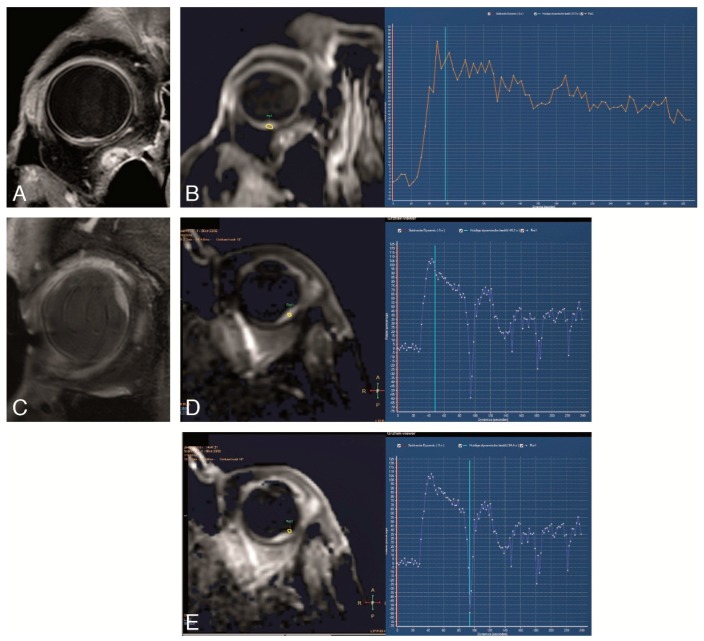
(**A**–**E**) DCE in two different patients. (**A**,**B**) Good DCE in a small tumor, with few movement artefacts and a wash-out TIC pattern. (**C**–**E**) DCE in a small uveal melanoma. Although with movement artefacts, these are possible to recognize by comparing the positioning of the ROI at the source images with the timepoint at the curve and noticing that the outliers in the curve match with ROI’s that due to eye movement are located outside the tumor. Also, with a wash-out TIC pattern. Notice the intraocular lens.

The evaluation of the PWI showed mostly a good quality and a wash-out TIC pattern. Eye motion and associated misregistration artefacts could compromise the evaluation of smaller tumors, but the comparison of the positioning of the region of interest (ROI) at the source images with the timepoint at the TIC curve made it possible to recognize these misregistration artefacts (Figure 9).

**Figure 10:**
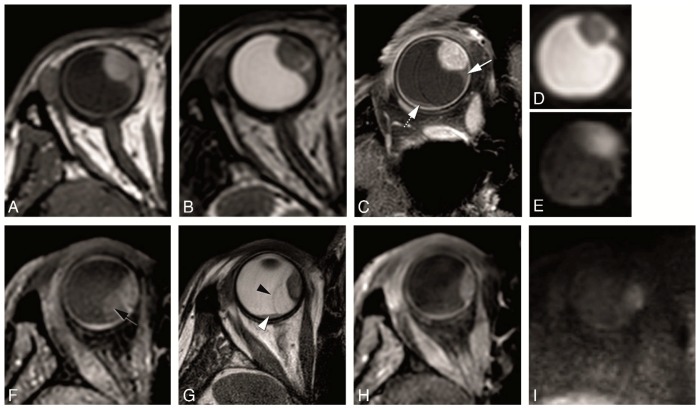
(**A**–**I**) Retinal detachment in two different patients. (**A**–**E**) MS 1 mm axial T1 (**A**), MS 1 mm axial T2 (**B**), MS 2 mm enhanced axial oblique T1 with fat signal suppression (**C**), DWI with b values of 0 s/mm^2^ (**D**) and 800 s/mm^2^ (**E**). Small homogeneous retinal detachment (arrow) located adjacent to the tumor, but also posterior and temporal (dashed arrow). It is distinguished from the tumor due to no enhancement, no diffusion restriction and a lentiform shape. (**F**–**I**) MS 1 mm axial T1 with fat signal suppression (**F**), MS 2 mm axial T2 (**G**), MS 1 mm enhanced axial T1 with fat signal suppression (**H**) and DWI with b value of 800 s/mm^2^ (**I**). Large heterogeneous retinal detachment. One bigger component which is better seen on T1 without contrast (black arrow), while on T2 only the retina being seen (black arrowhead) and the content being similar to the vitreous. One smaller component better seen on T2 (arrowhead), and due to being hemorrhagic hyperintense on T1 and with slight diffusion restriction.

The presence of retinal detachment was easy to appreciate and to differentiate from the UM due to lack of enhancement, its typical configuration and attachment at the optic nerve, and the absence of restriction diffusion (unless hemorrhagic) (Figure 10).

**Figure 11:**
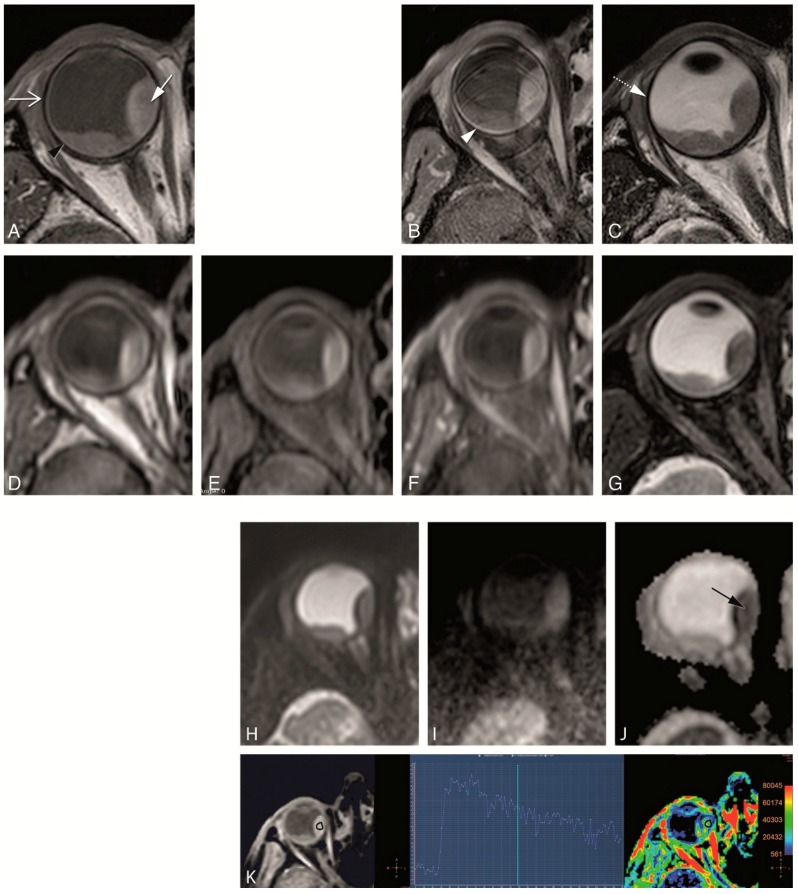
(**A**–**K**) Clinical protocol with anatomical and functional sequences in a patient with a uveal melanoma of the right eye (arrow). (**A**) MS 2 mm T1. (**B**) MS 2 mm enhanced T1 with fat signal suppression. (**C**) MS 2 mm T2. (**D**) 3D TSE 1 mm T1. (**E**) 3D TSE 1 mm T1 with fat signal suppression. (**F**) 3D TSE 1 mm enhanced T1 with fat signal suppression. (**G**) 3D TSE 0.8 mm T2 with fat signal suppression. (**H**–**J**) TSE DWI, with b values of 0 (**H**), 800 (**I**) and ADC (**J**). Restricted diffusion in the uveal melanoma (black arrow). (**K**) DCE with a good quality, few motion artefacts, showing a wash-out TIC pattern. Notice that on T2 WI the globe wall appears as a hypointense line (dashed arrow) and the different layers cannot be separated. On T1 the outer hypointense line corresponds to the sclera (open arrow), while the inner hyperintense layer corresponds to both the choroid and retina (black arrowhead). After contrast only the choroid and retina enhance (arrowhead). Anteriorly the ciliary body and iris can also be identified. The aqueous humor and vitreous body have a signal intensity similar to water. On the contrary, the lens is hypointense on T2 and slightly hyperintense on T1.

After evaluation of the extensive Study Protocol, a shorter Clinical Protocol was designed and evaluated in three UM patients. This clinical protocol consisted of MS 2 mm 2D sequences and a TSE DWI (b values of 0 and 800 s/mm^2^) and ADC, acquired perpendicular to the main axis of the tumor. It also included isotropic 3D TSE sequences and a DCE sequence, acquired on the axial plane non-angulated (Figure 11). The total scan time is 24 min. Notice that the MS pre-contrast T1 1 mm with fat signal saturation sequence was removed from the final protocol. Therefore, to continue with the same pre-contrast, as the post-contrast T1 sequence, which is with fat signal saturation, a 3D TSE T1 with fat signal saturation was added to the Clinical Protocol (Table 2).

## 4. Discussion

MRI of UM can be important for the diagnosis, choice of treatment, radiotherapy planning, potentially can predict treatment response and prognosis, and it should be able to early assess tumor response to radiotherapy. It is important to have a dedicated MRI protocol for the evaluation of uveal melanomas.

### 4.1. General Technical Requirements for Ocular MRI

Ocular imaging requires high spatial resolution, high contrast and an adequate signal-to-noise ratio. Image quality will depend on the coil and MRI sequences used and on the presence of eye motion artefacts, the latter also related with scan time.

Although a head coil can be used, optimal evaluation of the globe and therefore of UM is achieved by use of a surface coil due to the higher resolution and SNR obtained. Surface coils are less suitable for evaluating the deeper aspect of the orbit and the remainder of the visual pathway. However, visualizing the deeper orbit is not required routinely in the context of UM, since even extrascleral extension is usually limited to the tissues in close vicinity to the globe and can be evaluated with a surface coil. In the current setup a single coil is used to receive the MRI signal. The use of a multi-element receive coil would allow for a significant reduction on imaging time as the current images have sufficient SNR for acceleration through parallel imaging techniques such as GRAPPA (GeneRalized Autocalibrating Partially Parallel Acquisition) or SENSE (SENSitivity Encoding) [19,20]. However, at present such a multi-element coil is not available for clinical 3T MRI.

The eye has frequent voluntary and involuntary movements, both seriously degrading the MR images. Several methods have been described to minimize eye motion during the ocular MRI examination, including performing the MRI under retrobulbar anesthesia [21,22]. Although the application of retrobulbar anesthesia is effective in preventing eye motion, it is considered a too invasive procedure for a regular clinical setting. In our study, patients were only instructed to keep their eyes closed during scanning and images devoid or with few artefacts could be obtained. The longer the sequence the bigger the chance of eye motion artefacts and so imaging time should be kept to the minimum needed to ensure quality. In our study, except for the dynamic sequence, sequences lasted a maximum of 3 min and 35 s.

Another challenge of imaging the eye includes the location of the eye close to the air-tissue and bone-tissue interfaces and thus dealing with severe magnetic field inhomogeneity inducing distortion and signal dropouts. These magnetic susceptibility effects are amplified when using gradient echo as opposed to spin echo sequences. We also noticed that in particular the GE sequences are not good for the differentiation of the various layers of the globe wall and to identify the outer limit of the sclera and were therefore removed from the Clinical Protocol.

### 4.2. Anatomical MRI of Uveal Melanoma

The MRI protocol to evaluate an UM should include conventional sequences such as T1 and T2-WI sequences with and without a fat suppression and T1-WI sequences with a fat suppression technique after contrast medium administration. These are important for lesion characterization at diagnosis and in pretreatment evaluation. To interpret these images the radiologist needs to be acquainted with the characteristics of UM on MRI. Moreover, he/she should be familiar with the normal aspect of the various eye structures on MRI (Figure 11).

UM is usually hyperintense on T1, hypointense on T2 and it enhances. It is a uveal-based lesion, either with a lentiform, a dome or a mushroom-shape. The chemical shift artefact at the sclera-fat interface, is due to the different resonance frequency of protons in fat and water and results in a spatial displacement of the fat signal in the frequency-encoding direction, while the water signal from the sclera remains correctly placed. It is as though the fat image was cut out, moved a few pixels, and pasted on the background image. On image it appears as a signal void crescent area just adjacent and merging with the sclera. The location of this artefact depends on the frequency-encoding direction, usually chosen to be outside and not inside the globe because that would interfere with the evaluation of the UM. The radiologist needs to be acquainted with the chemical shift artefact at the sclera-orbital fat not to misinterpret it as sclera, which could result in an overestimation of the sclera thickness. The size of the water-fat shift depends on multiple scan parameters, including frequency-encoding gradient strength, resolution and magnetic field strength interface and it should be reduced to a minimum. In our sequences, we noticed that the water-fat shift artefact is usually more prominent on the MS than on the 3D sequences.

Tumor measurements are crucial for choosing adequate treatment, in particular to decide between enucleation and an eye-preserving therapy. Moreover, tumor measurements are important for the planning of the brachytherapy [3], as the plaque size used and the duration of treatment depend strongly on the size of the tumor. UM has been traditionally evaluated with ultrasound, but US tends to overestimate the tumor size [3,9]. The two important measurements that need to be taken are the tumor prominence and the largest basal tumor diameter. For accurate measurements, the spatial resolution of the sequences should be high enough to clearly identify the limits of the tumor, but also the outer limit of sclera, as the sclera thickness is also included when assessing the tumor prominence. The tumor prominence and the LBD need to be measured perpendicular and parallel to the main axis of the tumor, respectively. The measurement in one or two standardized planes can lead to considerable error in measuring the true tumor height, being the maximum error resulting from measurement in one plane as high as 1.41 times the true size [3]. To avoid this problem isotropic sequences with subsequent reconstructions should be performed. Also, three-dimensional data on the tumor geometry can be used for a more precise planning of the radiotherapy. In our study, several isotropic sequences were evaluated, all permitting multiplanar reconstructions. 3D reconstructions from the MS 1 mm sequences were frequently of suboptimal quality, showing stripes and wall deformation in case of motion. On the contrary, the 3D reconstructions of both the 3D TSE and 3D TFE sequences were mostly of good quality and less vulnerable to geometric distortions resulting from eye motion. Measurements should be preferably made on the 3D TSE enhanced T1 with fat signal suppression, since with this sequence good multiplanar reconstructions were possible, tumor is well differentiated from retinal detachment, the outer limit of the sclera is clearly identified, and no water-fat shift artefact is present. In case of no retinal detachment the 3D TSE T2 with fat signal suppression could also be used.

The evaluation of the extension of an UM, in particular whether extrascleral extension is present, is also crucial for treatment planning, since the presence of larger extrascleral extension generally implies enucleation. Extrascleral extension is present in 7% of the cases [10]. MRI is a valuable method for the assessment of scleral invasion and extrascleral extension, and is superior to US. In our study, the sequences best suited for that purpose were the MS 2 mm 2D sequences, which are acquired perpendicular to the tumor, because they were where the tumor limits and the different layers of the globe were best identified.

Retinal detachment is seen in 65.5% patients [10] and is regarded as a sign of progression of disease. The tumor needs to be differentiated from retinal detachment. This is especially important in a bid not to overestimate the maximal basal diameter. Retinal detachment was better recognized after contrast because retinal detachment does not enhance, and tumors do enhance. Others clues for this differentiation were that retinal detachment has a lentiform shape and a typical V shape with the vertex at the optic nerve [23]. 

### 4.3. Functional MRI of Uveal Melanoma

The MRI protocol to evaluate an UM should include functional images, such as DWI and PWI, which seem to be useful, although few studies have been published in UM [5,6,24,25].

DWI helps in distinguishing benign from malignant lesions, which is important in the differential diagnosis of UM [26]. It also helps in the differentiation between UM and retinal detachment, with UM showing diffusion restriction and with retinal detachment, except when hemorrhagic, with no diffusion restriction. Moreover, DWI seems effective on the pretreatment prediction of treatment outcome, with low ADC being correlated with good response and high ADC with poor response [5]. Finally, in UM treated with proton-beam therapy, ADC variations precede volume changes and early change in ADC value 1 month after therapy significantly correlated with tumor regression [5]. Although generally EPI techniques are used for DWI, the TSE technique is preferable for the orbit as it is less susceptible for the present magnetic field inhomogeneities [27]. Our images showed that this non-EPI technique results in good SNR, good contrast and almost no distortion. We achieved images where, even for tumors as small as 2 mm, restricted diffusion could be appreciated. We found no added value of the DWI images with a b value of 400 s/mm^2^ and therefore it was removed from our Clinical protocol.

PWI provides data in the wash-in and wash-out contrast kinetics within a lesion. In DCE-MRI the qualitative evaluation of the TIC pattern seems to be a complementary investigation in distinguishing benign from malignant lesions. In the study from Yuan et al. [24], a persistent TIC pattern (type I curve) suggests a benign lesion, a wash-out TIC pattern (type III curve) mostly suggests malignancy, and a plateau TIC pattern (type II curve) occurs both in benign and malignant lesions [24,27]. Additionally, PWI seems to give prognostic information, in the study from Kamrava et al., with a significant correlation between the k^trans^ and percent of monossomy 3 > 33% [6]. Finally, PWI seems to be useful in the follow-up of UM treated with episcleral brachytherapy [28]. Most PWI in the orbit and globe used a DCE technique in which serial T1-weighted images are acquired before, during and after contrast administration. In our study, we also used a DCE technique. The challenge of studying the perfusion of a globe lesion is mainly related to eye motion with its associated misregistration artefacts, but in our study the DCE images were mostly diagnostic. The presence of eye motion is easy to check by looking at the source images from the perfusion sequence. Eye motion could compromise the evaluation of small UM, where the ROI drawn in the tumor to obtain a time intensity curve would, due to eye motion, fall outside the tumor and in the TIC appear as an outlier. The comparison of the positioning of the ROI at the source images with the timepoint at the TIC curve makes it possible to recognize these misregistration artefacts, obviating this problem.

To determine the optimal MRI protocol, a relatively large number of scans had to be compared with each other. As a result, the study protocol takes about an hour to acquire, which is about twice as long as a regular clinical MRI scan. Because of that, the study protocol is quite a burden for the patients. Since the MRI images of the nine included patients gave a consistent picture of the optimal scans for high-quality MRI scans of the eye, this relatively small group of patients was sufficient to determine the final clinical protocol. The group of nine included patients is, however, too small to evaluate the clinical value of MRI for UM, but this evaluation can be made in subsequent studies using the proposed clinical protocol, which takes approximately half an hour.

### 4.4. Clinical MRI Protocol for UM

The final clinical protocol developed includes MS 2 mm 2D sequences for lesion characterization and local extension evaluation. Isotropic 3D TSE sequences are also performed for accurate geometric measurements of the tumor, being therefore suitable for therapy planning. A TSE DWI sequence, with b values of 0 and 800 s/mm^2^, can be used to confirm the malignancy of the lesion and is furthermore proposed to provide an earlier biomarker for therapy response. Finally, the contrast-enhanced scans allow for proper differentiation between tumor and retinal detachment, while the DCE sequence is used to assess the tumor hemodynamics.

## 5. Conclusions

By combining a dedicated MRI protocol with a local receive eye coil, high-resolution MRI images of UM can be obtained. This multiparametric MRI should ideally include 2D sequences for the diagnosis and determination of tumor extension, and isotropic 3D sequences for therapy planning. Furthermore, DWI and PWI sequences could be included to aid differential diagnosis, potentially giving prognostic information and important for the follow-up after radiotherapy.

## Figures and Tables

**Table 1:** Patients’ data regarding sex, eye involved, treatment, histology in case of enucleation and classification according to the AJCC. OD—oculus dexter, OS—oculus sinister**,** PBT—proton-beam therapy**,** AJCC—American Joint Committee on Cancer.

Patients	Sex	Eye	Treatment	Histology	Classification (AJCC)
**1**	Female	OD	PBT		T3b
**2**	Male	OD	Enucleation	Melanoma	T2b
**3**	Male	OD	Brachytherapy		T1a
**4**	Male	OD	Brachytherapy		T1a
**5**	Male	OS	Brachytherapy		T2a
**6**	Female	OD	Enucleation	Melanoma	T4b
**7**	Male	OD	Brachytherapy		T3a
**8**	Male	OD	Enucleation	Melanoma	T4a
**9**	Male	OD	Enucleation	Melanoma	T3a

**Table 2:** Scans’ parameters of the anatomical and functional sequences from the Study Protocol and of the 3D TSE T1 SPIR, the latter not part of the Study Protocol, but lately added to the Clinical Protocol. Both the multi-slice (MS) 2 mm and the diffusion-weighted imaging sequences are planned perpendicular to the tumor, using the 3D scans as a reference. During the Dynamic Contrast-Enhanced (DCE) scan, the contrast agent is administered and afterwards the contrast-enhanced (Gd) scans are acquired.

Purpose	Scan Name	Voxel Size (mm^3^)	FOV (mm^3^)	Oversampling (mm)	Echo Train Length	TE(ms)/TR(ms)/Flip or ref. Angle (deg)	Fat Supr.	Avg.	Scan Time (mm:ss)	Additional Parameters
3D measurements	MS TSE 1 mm T1	0.9 × 0.9 × 1.0	80 × 80 × 40	70 mm	8	8.0/718/180	-	1	02:35	
	MS TSE 1 mm T1 SPIR	0.9 × 0.9 × 1.0	80 × 80 × 40	60 mm	6	8/636/180	SPIR	1	02:27	
	MS TSE 1 mm T2	0.9 × 0.9 × 1.0	80 × 80 × 40	20 mm	17	90/4436/120	-	2	02:04	
	3D TSE T1	1.0 × 1.0 × 1.0	80 × 80 × 40	40 mm	14	9.4/350/180	-	1	03:23	
	3D TSE T1 SPIR	1.0 × 1.1 × 1.0	80 × 80 × 40	45 mm	14	9.4/350/180	SPIR	1	03:23	
	3D TSE T2 SPIR	0.8 × 0.8 × 0.8	50 × 81 × 40	4 REST slabs	117	293/2500/35	SPIR	2	03:35	
	3D TFE T1	0.8 × 0.8 × 0.8	80 × 80 × 40	4 REST slabs	100	2.5/5/10	-	1	03:21	T_inv_: 1000 ms
	MS TSE 1 mm T1 SPIR Gd	0.9 × 0.9 × 1.0	80 × 80 × 40	60 mm	6	8/636/180	SPIR	1	02:27	
	3D TSE T1 SPIR Gd	1.0 × 1.1 × 1.0	80 × 80 × 40	45 mm	14	9.4/350/180	SPIR	1	03:23	
	3D TFE T1 PROSET Gd	0.8 × 0.8 × 0.8	80 × 80 × 40	4 REST slabs	100	4.8/8.4/10	Proset 1331	1	03:22	
Tumor origin & extension	MS TSE 2 mm T1	0.5 × 0.5 × 2.0	100 × 100 × 24	-	6	8/718/180	-	1	01:16	
	MS TSE 2 mm T1 SPIR Gd	0.5 × 0.5 × 2.0	100 × 100 × 24	-	6	80/764/180	SPIR	1	01:16	
	MS TSE 2 mm T2	0.4 × 0.4 × 2.0	100 × 100 × 24	-	17	90/1331/120	-	2	01:25	
Functional scans	DWI (TSE)	1.25 × 1.4 × 2.4	100 × 100 × 24	24 mm	single shot	64/5759/50	SPIR	5	03:21	B = 0, 400, 800 s/mm^2^
	DCE	1.25 × 1.5 × 1.5	80 × 80 × 32			2.3/4.5/5	Proset 11	1	04:20	2 sec/dynamic

**Table 3:** Comparison of the anatomical sequences. ++ very good, + good, +− sufficient, − insufficient, RD—retinal detachment.

Evaluated Parameters	Isotropic Sequences									2D Sequences Perpendicular to the Tumor—MS 2 mm		
	T2-Weighted		T1-Weighted Before Contrast				T1-Weighted After Contrast					
	MS TSE	3D TSE SPIR	MS TSE	MS TSE SPIR	3D TSE	3D TFE	MS TSE SPIR	3D TSE SPIR	3D TFE PROSET	MS T2	MS T1	MS T1 SPIR Gd
**In-plane image quality**												
General image quality	++	+	+	+	+−	+−	+	+−	+	+	+	++
Contrast	+	++	+−	+	+−	+	++	+	++	++	+	++
Outer limit of sclera	++	+	+	+	+	−	+	+	−	++	++	+
Outer limits of tumor	+−	+−	+	+	+−	−	++	+	−	+	+	+
Differentiate tumor vs RD							++	++	+			++
**3D analysis**												
Geometrical accuracy												
Good of complete eye	75%	78%	44%	44%	77%	100%	50%	88%	100%			
Small local artefacts	25%	22%	33%	55%	11%	0%	25%	13%	0%			
Not usable	0%	0%	22%	0%	11%	0%	25%	0%	0%			
Outer limit of sclera	++	+	+−	+−	+	−	+−	+	−			
